# Prevalence of atherosclerosis in individuals with prediabetes and diabetes compared to normoglycaemic individuals—a Swedish population-based study

**DOI:** 10.1186/s12933-023-01982-6

**Published:** 2023-09-27

**Authors:** Carl Johan Östgren, Julia Otten, Karin Festin, Oskar Angerås, Göran Bergström, Kerstin Cederlund, Gunnar Engström, Maria J. Eriksson, Mats Eriksson, Tove Fall, Anders Gummesson, Emil Hagström, Urban Hellman, Stefan K. James, Tomas Jernberg, Johan Kihlberg, David Kylhammar, Hanna Markstad, Peter Nilsson, Anders Persson, Margaretha Persson, Carlo Pirazzi, Rebecka Renklint, Annika Rosengren, Stefan Söderberg, Johan Sundström

**Affiliations:** 1 Department of Health, Medicine and Caring Sciences, Centre of Medical Image Science and Visualization (CMIV), Linköping University, 581 83 Linköping, SE Sweden; 2 Department of Health, Medicine and Caring Sciences, Linköping University, Linköping, Sweden; 3 Department of Public Health and Clinical Medicine, Section of Medicine, Umeå University, Umeå, Sweden; 4 Department of Cardiology, Sahlgrenska University Hospital, Gothenburg, Sweden; 5 Department of Molecular and Clinical Medicine, Institute of Medicine, Sahlgrenska Academy, University of Gothenburg, Gothenburg, Sweden; 6 Clinical Physiology, Sahlgrenska University Hospital, Gothenburg, Sweden; 7 Department of Clinical Science, Intervention and Technology, Karolinska Institute, Stockholm, Sweden; 8 Department of Clinical Sciences in Malmö, Lund University, Malmö, Sweden; 9 Department of Molecular Medicine and Surgery, Karolinska Institute, Stockholm, Sweden; 10 Department of Clinical Physiology, Karolinska University Hospital, Stockholm, Sweden; 11 Medicine Unit Endocrinology, Theme Inflammation and Ageing, Karolinska University Hospital, Stockholm, Sweden; 12 Unit of Endocrinology, Department of Medicine, Karolinska Institute at Karolinska University Hospital Huddinge, Stockholm, Sweden; 13 Department of Medical Sciences, Molecular Epidemiology, Uppsala University, Uppsala, Sweden; 14 Department of Clinical Genetics and Genomics, Sahlgrenska University Hospital, Region Västra Götaland, Gothenburg, Sweden; 15 Department of Medical Sciences, Cardiology, Uppsala University, Uppsala, Sweden; 16 Uppsala Clinical Research Center, Uppsala University, Uppsala, Sweden; 17 Department of Clinical Sciences, Danderyd University Hospital, Karolinska Institute, Stockholm, Sweden; 18 Department of Radiology and Department of Health, Medicine and Caring Sciences, Linköping University, Linköping, Sweden; 19 Division of Diagnostics and Specialist Medicine, Department of Health, Medicine and Caring Sciences and Department of Clinical Physiology, Linköping University, Linköping, Sweden; 20 Center for Medical Imaging and Physiology, Skåne University Hospital and Lund University, Lund, Sweden; 21 Experimental Cardiovascular Research, Clinical Research Center, Clinical Sciences Malmö, Lund University, Malmö, Sweden; 22 Department of Internal Medicine, Skåne University Hospital, Malmö, Sweden; 23 Department of Clinical Sciences, Huddinge University Hospital, Karolinska Institute, Stockholm, Sweden; 24 Department of Public Health and Clinical Medicine, Umeå University, Umeå, Sweden; 25 Department of Medicine, Geriatrics and Emergency Medicine, Sahlgrenska University Hospital Östra Hospital, Gothenburg, Sweden; 26 Department of Medical Sciences, Uppsala University, Uppsala, Sweden; 27 The George Institute for Global Health, University of New South Wales, Sydney, Australia

**Keywords:** Atherosclerosis, Carotid arteries, Coronary arteries, Coronary computed tomography angiography, Diabetes, Prediabetes

## Abstract

**Background:**

Patients with type 2 diabetes have an increased risk of death and cardiovascular events and people with diabetes or prediabetes have been found to have increased atherosclerotic burden in the coronary and carotid arteries. This study will estimate the cross-sectional prevalence of atherosclerosis in the coronary and carotid arteries in individuals with prediabetes and diabetes, compared with normoglycaemic individuals in a large population-based cohort.

**Methods:**

The 30,154 study participants, 50–64 years, were categorized according to their fasting glycaemic status or self-reported data as normoglycaemic, prediabetes, and previously undetected or known diabetes. Prevalence of affected coronary artery segments, severity of stenosis and coronary artery calcium score (CACS) were determined by coronary computed tomography angiography. Total atherosclerotic burden was assessed in the 11 clinically most relevant segments using the Segment Involvement Score and as the presence of any coronary atherosclerosis. The presence of atherosclerotic plaque in the carotid arteries was determined by ultrasound examination.

**Results:**

Study participants with prediabetes (n = 4804, 16.0%) or diabetes (n = 2282, 7.6%) had greater coronary artery plaque burden, more coronary stenosis and higher CACS than normoglycaemic participants (all, p < 0.01). Among male participants with diabetes 35.3% had CACS ≥ 100 compared to 16.1% among normoglycaemic participants. For women, the corresponding figures were 8.9% vs 6.1%. The prevalence of atherosclerosis in the coronary arteries was higher in participants with previously undetected diabetes than prediabetes, but lower than in patients with known diabetes. The prevalence of any plaque in the carotid arteries was higher in participants with prediabetes or diabetes than in normoglycaemic participants.

**Conclusions:**

In this large population-based cohort of currently asymptomatic people, the atherosclerotic burden in the coronary and carotid arteries increased with increasing degree of dysglycaemia. The finding that the atherosclerotic burden in the coronary arteries in the undetected diabetes category was midway between the prediabetes category and patients with known diabetes may have implications for screening strategies and tailored prevention interventions for people with dysglycaemia in the future.

## Background

Patients with type 2 diabetes have a two to four times higher risk of death and cardiovascular events than the general population [1]. Type 2 diabetes is usually preceded by a prediabetic state characterised by elevated blood glucose levels, i.e., impaired fasting glucose (IFG) or impaired glucose tolerance (IGT), both of which carry an increased risk of cardiovascular disease [2].

From previous studies in this field, we know that an increased coronary artery calcium score (CACS) and atherosclerosis in the carotid arteries are present in diabetes and prediabetes [3–7]. With modern imaging technology [8], it is possible to visualize atherosclerotic plaques non-invasively using coronary computed tomography angiography (CCTA) [9] to identify individuals with subclinical coronary artery disease.

The prevalence of atherosclerosis in currently asymptomatic individuals with prediabetes, and particularly in those with previously undetected diabetes, has not been extensively studied. Understanding the extent of atherosclerosis in people with prediabetes and diabetes compared to normoglycaemic individuals may be useful for future screening strategies and tailored preventive interventions.

The aim of the current study was to determine the cross-sectional prevalence of asymptomatic atherosclerosis in the coronary and carotid arteries and of indicators of peripheral artery disease in individuals with previously undetected or known diabetes and prediabetes compared with normoglycaemic individuals. This was done using the large population-based Swedish CArdioPulmonary bioImage Study (SCAPIS), using CCTA to determine the extent and characteristics of coronary artery atherosclerosis [10]. The presence of atherosclerosis in the carotid arteries was determined by ultrasound and asymptomatic peripheral artery disease was determined by the ankle-brachial index (ABI).

## Methods

### Study population

**Fig. 1:**
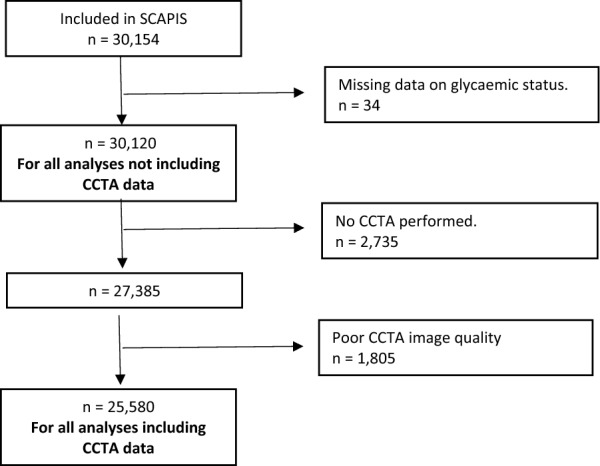
Flowchart of study population. *CCTA* coronary computed tomography angiography

SCAPIS is a general population-based cohort study (www.scapis.org). SCAPIS enrolled randomly recruited study participants from the census register aged 50–64 years (51% women) at six university hospitals in Sweden during 2013–2018. History of cardiovascular disease was defined as previous myocardial infarction, coronary artery bypass grafting, or percutaneous coronary intervention, either self-reported (in the study questionnaire) or provided by at least one previous diagnosis of myocardial infarction or cardiac intervention in the Swedish inpatient register (National Board of Health and Welfare), as previously described [10]. The participation rate was 50% of the invited population. A flowchart of the study population is illustrated in Fig. 1.

The SCAPIS study was approved by the regional ethics committee in Umeå (Dnr 2010-228-31 M) and complied with the Declaration of Helsinki. Written informed consent was obtained from all participants.

### Study procedures

The procedures of the study have previously been described in detail [10, 11]. In the present study, we used data from cardiac imaging, carotid ultrasound, physical examinations, routine laboratory tests and a comprehensive questionnaire. After an overnight fast, a venous blood sample was taken for analysis of plasma glucose, HbA1c, lipids, creatinine and high-sensitivity C-reactive protein (CRP).

### Glycaemic status

The study participants were categorised according to their one single occasion fasting glycaemic status: normoglycaemic [glucose: < 6.1 mmol/L and HbA1c < 6.0% (< 42 mmol/mol)], pre-diabetes (6.1–6.9 mmol/L and/or elevated HbA1c 6.0–6.5% [42–47 mmol/mol)], previously undetected diabetes (glucose ≥ 7.0 mmol/L and/or HbA1c ≥ 6.5% (≥ 48 mmol/mol)] or self-reported known diabetes [12, 13]. Prediabetes can be defined by either IGT, IFG or an elevated HbA1c level. In this study, we used both IFG and an elevated HbA1c value to define prediabetes, as suggested by the recommendations of NICE [12].

### Cardiac imaging

Cardiac imaging in SCAPIS has already been described in detail [10]. Briefly, coronary artery calcification was assessed in non-contrast-enhanced images from a multi-slice computed tomography scanner (Siemens, Somatom Definition Flash, Siemens Healthineers, Erlangen, Germany). Imaging and analyses were performed using dedicated software for calcium scoring and coronary artery assessment. The calcium content in each coronary artery was measured and summed to give an overall CACS according to international standards.

**Fig. 2:**
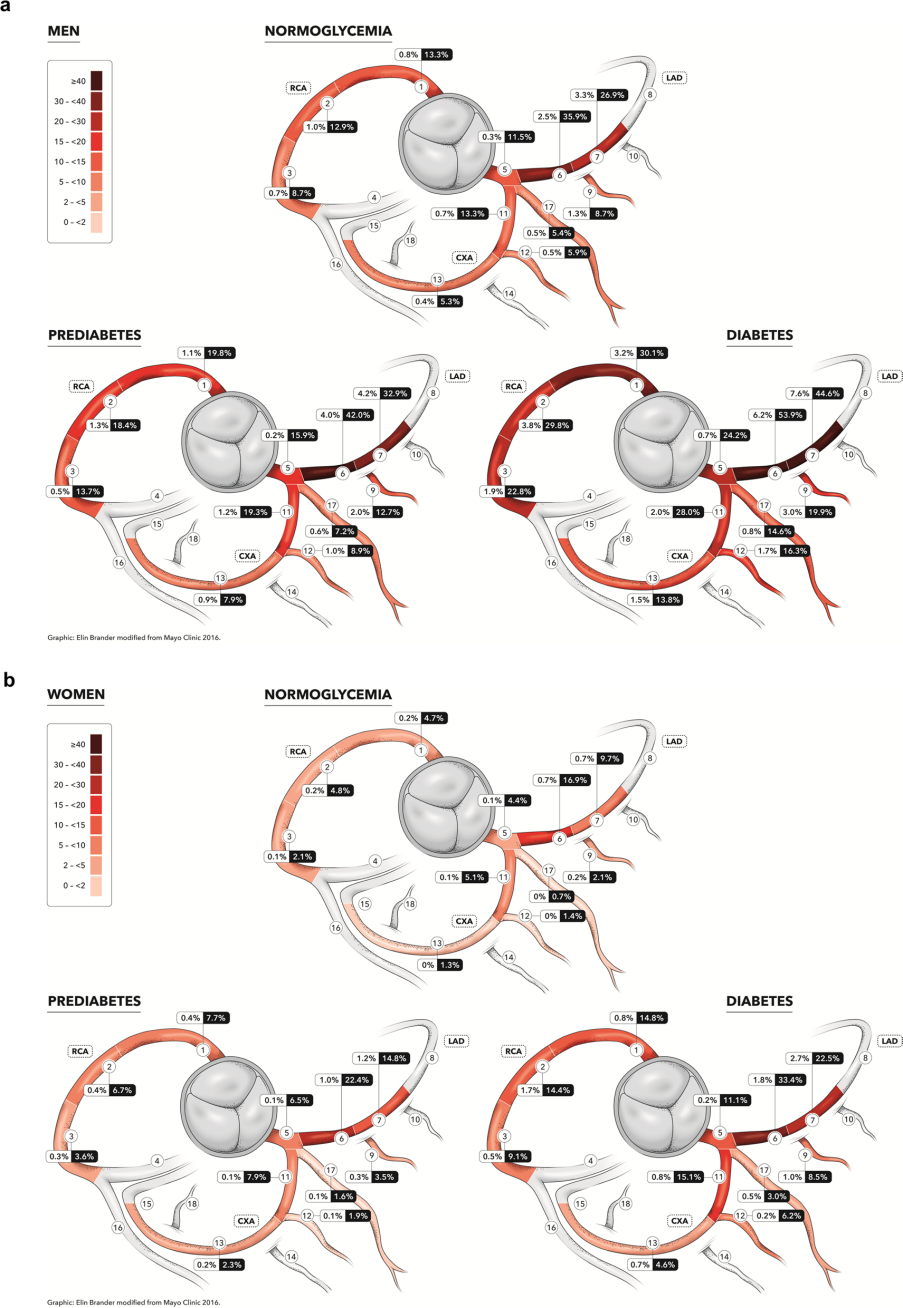
**a**. Distribution of CCTA-detected atherosclerosis in SCAPIS male participants according to glycaemic status. Frequency of atherosclerosis in the 11 most proximal coronary segments in men in the SCAPIS cohort. The heat map refers to frequency of any form of atherosclerosis. The number within boxes refers to the coronary stenosis status (≥ 50% /any atherosclerosis). **b**. Distribution of CCTA-detected atherosclerosis in SCAPIS female participants according to glycaemic status. Frequency of atherosclerosis in the 11 most proximal coronary segments in women in the SCAPIS cohort. The heat map refers to frequency of any form of atherosclerosis. The number within boxes refers to the coronary stenosis status (≥ 50%/any atherosclerosis)

For reporting coronary atherosclerosis from CCTA, we used the 18 coronary segment model defined by the Society of Cardiovascular Computed Tomography [14].To streamline reading and increase quality of the most important findings, readers focused on the 11 clinically most relevant segments (segments 1 through 3, 5 through 7, 9, 11 through 13, and 17), which were compulsory to report; the remaining segments were only reported if they had atherosclerosis or calcium blooming [10]. Coronary vessel status per segment was defined as: no atherosclerosis; 1–49% stenosis; >  = 50% (i.e., significant) stenosis. Segments with non- calcified plaques were also identified. The percentage frequency is given for significant stenosis (≥ 50%) and any atherosclerosis (Fig. 2a, b). Total coronary artery atherosclerotic burden was calculated using the Segment Involvement Score (SIS), a measure of the total number of coronary segments with atherosclerosis, regardless of the degree of stenosis. A SIS ≥ 4 has previously been associated with worse cardiovascular outcomes [15].

### Carotid artery plaque

Atherosclerosis in the carotid arteries was determined according to a standardized protocol using a Siemens Acuson S2000 ultrasound machine with a 9L4 linear transducer (both from Siemens Healthineers, Erlangen, Germany). The left and right carotid arteries were isolated and atherosclerotic plaques were identified in the common carotid artery, the bulb or the internal carotid artery according to the Mannheim consensus [16]. Accordingly, plaques were defined as focal structures penetrating at least 0.5 mm or 50% of the surrounding intima-media thickness into the arterial lumen or having a thickness > 1.5 mm, measured from the intima-lumen boundary to the media adventitia boundary. Carotid atherosclerosis was defined by ≥ 1 plaque in one or both carotid arteries.

### Ankle–brachial index

Arterial blood pressure at the ankles was measured bilaterally with a Doppler pulse sensor while the subject was supine. Systolic blood pressure was measured in the dorsalis pedis artery and posterior tibial artery with a blood pressure cuff on the lower calf. The ABI was calculated for each ankle artery as the ratio between the systolic ankle pressure and the systolic brachial artery pressure. Two measurements were taken at each ankle artery until stable pressures were achieved (within ± 10 mmHg). Peripheral artery disease was defined by an ABI of less than 0.9.

### Statistical methods

**Fig. 3:**
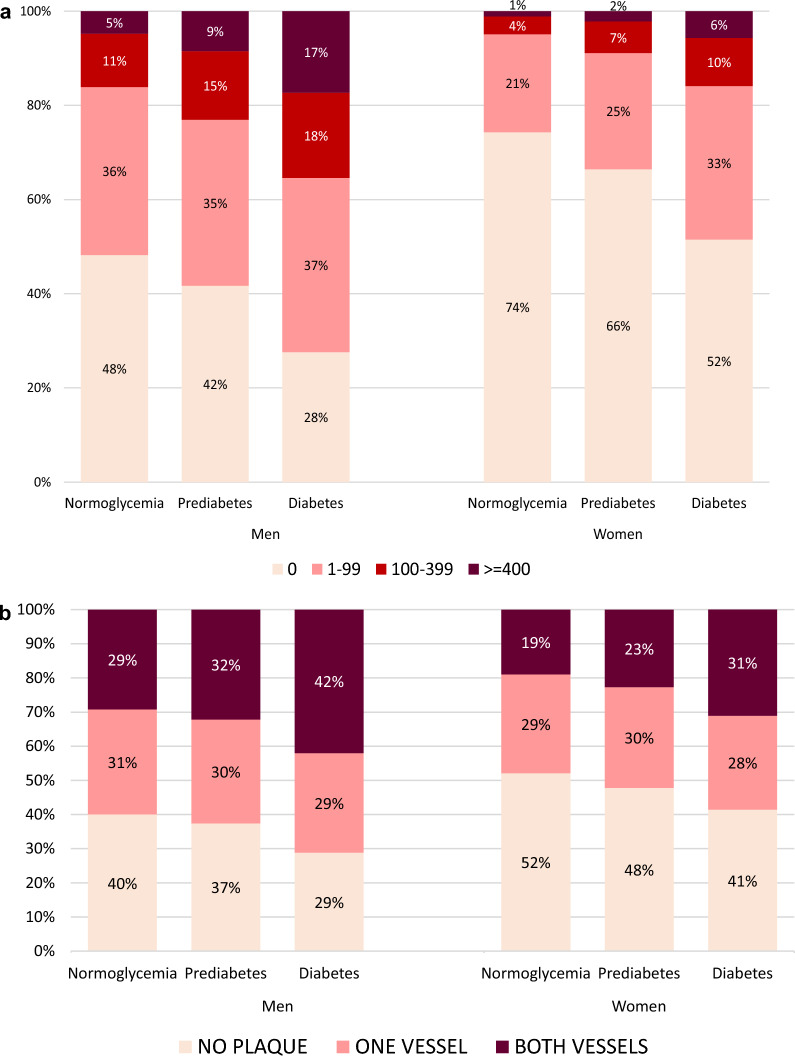
**a** Frequency of CACS categories, by glycaemic status as in Fig. 2 in SCAPIS participants. **b** Frequency of plaque in carotid arteries, by glycaemic status as in Fig. 2 in SCAPIS participants

**Fig. 4:**
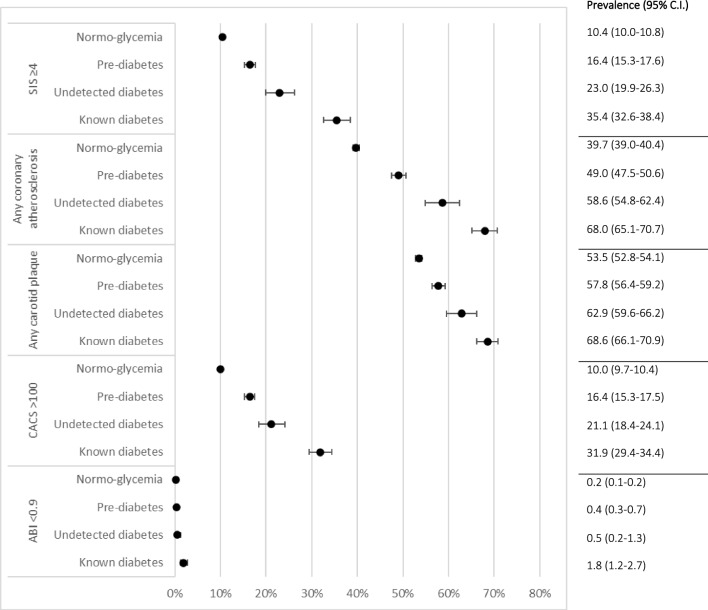
Prevalence of coronary computed tomography angiography–detected atherosclerosis, ultrasound detected plaque in carotid arteries and ankle-brachial index in SCAPIS participants stratified by glycaemic status in four groups

Background factors for the population, stratified on sex and glycaemic status, was described descriptively (n, mean, SD, median, IQR) in Table 1. Prevalence (95% C.I.) of any form of atherosclerosis, stenosis ≥ 50%, any stenosis, SIS ≥ 4, carotid plaque, CACS > 100 and ABI < 0.9 were illustrated in Figs. 2, 3, 4. Differences in the prevalence of atherosclerosis between the four glycaemic status groups were analyzed using ordinal and logistic regression models adjusted for age, sex, smoking and site. To assess the association between subclinical atherosclerosis and glycaemic status, HbA1c level and diabetes duration, respectively, three outcomes were evaluated: CACS, carotid artery atherosclerosis and ABI. The CACS was divided into four groups (< 1, 1–99, 100–399, ≥ 400) and coronary vessel status per segment was defined as: no atherosclerosis; 1–49% stenosis; >  = 50% (i.e., significant) stenosis. Presence of plaques in the carotid arteries was divided into three groups: no plaque, plaque in one carotid artery or plaque in both carotid arteries. Any carotid artery plaque is hence defined as ≥ 1 plaque. ABI was dichotomized into < 0.9 resp. ≥ 0.9. A total of six ordinal and three logistic regression models were run where the three exposures were diabetes glycaemia (normal glycaemia as the reference group compared with prediabetes, undetected diabetes and known diabetes), HbA1c and years of diabetes duration. All regression models were adjusted for age, sex, pack-years of smoking, and site. Sensitivity analyses for these associations were performed, where individuals with previous myocardial infarction (MI), coronary artery bypass graft (CABG), percutaneous coronary intervention (PCI) were excluded (n = 693). Correction for possible false discovery rate due to multiple testing were done according to the method described by Benjamini and Hochberg [17]. Data were analyzed without imputations of missing data.

**Table 1:** Characteristics of SCAPIS participants stratified by sex and glycemic status

	Men				Women			
	Total	Normoglycaemia	Pre-diabetes	Diabetes	Total	Normoglycemia	Pre-diabetes	Diabetes
Sample size -n (%)	14624	10634 (72.7)	2571 (17.6)	1419 (9.7)	15496	12400 (80.0)	2233 (14.4)	863 (5.6)
Sociodemographics								
Age—mean (SD)	57.5 (4.4)	57.1 (4.3)	58.2 (4.3)	59.1 (4.2)	57.5 (4.3)	57.3 (4.3)	58.2 (4.3)	58.8 (4.0)
Education, highest level								
< Compulsory school (%)	100 (0.7)	59 (0.6)	23 (0.9)	19 (1.4)	95 (0.6)	58 (0.5)	26 (1.2)	11 (1.3)
Compulsory school (%)	1382 (9.9)	1734 (7.7)	497 (10.7)	318 (14.7)	1137 (7.6)	822 (6.8)	217 (10.0)	111 (13.4)
Upper Secondary Highschool (%)	6798 (48.6)	9971 (44.4)	2268 (48.8)	1083 (50.0)	6350 (42.5)	5075 (41.9)	954 (44.0)	404 (48.9)
University (%)	5712 (40.8)	10638 (47.7)	1834 (39.5)	733 (33.9)	7344 (49.2)	6170 (50.9)	973 (44.8)	300 (36.3)
History of cardiovascular disease								
Previous MI, CABG or PCI n (%)	541 (3.7)	254 (2.4)	154 (6.0)	129 (9.1)	152 (1.0)	74 (0.6)	37 (1.7)	41 (1.7)
Family history of cardiovascular diseases								
MI, subjects’ parent or sibling n (%)	3589 (26.2)	2561 (25.6)	629 (26.2)	399 (31.7)	4606 (31.5)	3618 (30.7)	702 (33.8)	286 (37.0)
Stroke subjects’ parent or sibling n (%)	3462 (25.5)	2467 (24.9)	654 (27.4)	332 (26.8)	4300 (29.6)	3428 (29.4)	612 (29.8)	260 (33.2)
Medication								
Antihypertensive agents n (%)	3030 (21.6)	1687 (16.5)	681 (27.7)	662 (49.6)	2733 (18.2)	1829 (15.2)	546 (25.4)	358 (43.6)
Lipid lowering agents n (%)	1386 (9.9)	640 (6.3)	317 (12.9)	429 (32.2)	911 (6.1)	480 (4.0)	182 (8.5)	249 (30.3)
Glucose lowering agents (oral only) n (%)	–	–	–	696 (52.3)	–	–	–	378 (46.4)
Insulin only or insulin + oral treatment				242 (30.4)				121 (25.8)
Anthropometry								
BMI, kg/m2 median (Q1–Q3)	26.9 (24.8–29.6)	26.4 (24.4–28.9)	27.9 (25.6–30.6)	29.3 (26.8–32.7)	25.7 (23.1–29.1)	25.1 (22.8–28.2)	27.7 (24.6–31.6)	30.0 (26.4–34.1)
Waist Circumference, cm median (Q1–Q3)	99.0 (92.0–106.0)	97.0 (91.0–104.0)	102.0 (96.0–110.0)	106.0 (99.0–115.0)	88.0 (80.0–97.0)	86.0 (79.0–95.0)	94.0 (85.0–103.0)	100.0 (91.0–110.0)
Life style								
Current smoking status								
Current	1191 (11.6)	1191 (11.6)	385 (15.6)	220 (16.5)	1432 (11.9)	1432 (11.9)	353 (16.4)	145 (17.7)
Ex-smoker (regardless of cessation time)	3302 (32.2)	3302 (32.2)	910 (37.0)	540 (40.4)	4623 (38.5)	4623 (38.5)	875 (40.7)	338 (41.2)
Never	5764 (56.2)	5764 (56.2)	1167 (47.4)	575 (43.1)	5956 (49.6)	5956 (49.6)	921 (42.9)	337 (41.1)
Pack-years—median (Q1–Q3)	13.0 (5.5–25.0)	12.0 (5.0–23.3)	15.0 (6.8–27.8)	18.0 (9.3–30.6)	12.0 (5.0–21.5)	11.1 (4.5–20.5)	13.8 (6.0–24.8)	18.5 (9.9–29.3)
Physical activity (min/day)								
Sedentary median (Q1–Q3)	180 (120–255)	182 (121–253)	187 (127–263)	203 (137–287)	151 (102–217)	151 (103–215)	154 (103–225)	174 (113–249)
Low-intensity median (Q1–Q3)	330 (272–393)	328 (273–389)	329 (272–394)	309 (254–373)	363 (307–422)	362 (308–423)	370 (309–431)	348 (286–414)
Moderate- and vigorous median (Q1–Q3)	49.0 (32.0–71.0)	54.0 (37.0–75.0)	52.0 (35.0–73.0)	43.0 (28.0–65.0)	48.0 (32.0–67.0)	51.0 (36.0–70.0)	47.0 (32.0–67.0)	41.0 (26.0–59.0)
Laboratory measurements								
Fasting glucose, mmol/l—mean (SD)	5.7 (1.3)	5.3 (0.5)	6.3 (0.4)	8.3 (2.5)	5.5 (1.0)	5.2 (0.5)	6.2 (0.5)	7.9 (2.4)
HbA1c, mmol/mol—mean (SD)	36.9 (7.2)	34.8 (3.1)	37.7 (4.0)	51.0 (14.5)	36.3 (5.7)	35.0 (3.0)	38.3 (4.1)	49.3 (14.1)
HbA1c, %	5.5	5.4	5.6	6.8	5.4	5.4	5.6	6.6
High sensitivity CRP, mg/L—mean (SD)	2.1 (4.6)	1.9 (3.8)	2.4 (4.5)	3.1 (8.6)	2.2 (4.1)	1.9 (3.7)	3.0 (5.3)	3.5 (4.8)
Total cholesterol, mmol/L—mean (SD)	5.32 (1.06)	5.45 (1.00)	5.13 (1.06)	4.65 (1.11)	5.64 (1.02)	5.70 (1.00)	5.52 (1.01)	5.08 (1.15)
HDL, mmol/L—mean (SD)	1.41 (0.40)	1.45 (0.39)	1.37 (0.38)	1.24 (0.37)	1.83 (0.50)	1.88 (0.49)	1.71 (0.45)	1.54 (0.50)
LDL, mmol/L—mean (SD)	3.43 (0.97)	3.56 (0.93)	3.27 (0.96)	2.84 (1.01)	3.45 (0.96)	3.49 (0.95)	3.39 (0.94)	3.02 (1.07)
Remnant, mmol/L—mean (SD)	0.47 (0.41)	0.46 (0.40)	0.49 (0.44)	0.57 (0.41)	0.36 (0.34)	0.33 (0.33)	0.43 (0.33)	0.50 (0.38
Triglycerides, mmol/L—mean (SD)	1.4 (1.0)	1.3 (0.9)	1.4 (1.2)	1.8 (1.4)	1.1 (0.6)	1.1 (0.5)	1.2 (0.7)	1.6 (1.1)
eGFR ml/min/1,73 m2 mean (SD)	85.3 (11.8)	84.7 (11.5)	85.9 (12.2)	88.3 (13.1)	84.4 (12.4)	84.1 (12.2)	85.2 (12.4)	87.1 (13.9)
eGFR, ml/min/1,73 m2 < 60 n (%)	299 (2.0)	190 (1.8)	58 (2.3)	51 (3.6)	409 (2.6)	325 (2.6)	54 (2.4)	30 (3.5)
Clinical characteristics								
Diabetes duration, years- mean (SD)	–	–	–	9.3 (9.1)	–	–	–	10.9 (11.7)
Systolic blood pressure, mmHg—mean (SD)	128.8 (15.6)	127.5 (15.4)	131.4 (15.5)	133.8 (16.1)	123.2 (17.8)	122.2 (17.7)	126.5 (17.7)	129.0 (16.8)
Diastolic blood pressure, mmHg—mean (SD)	78.5 (10.1)	78.0 (10.2)	79.8 (9.9)	80.2 (9.9)	76.6 (10.8)	76.2 (10.8)	78.3 (10.7)	78.8 (9.9)
Ankle-brachial index—median (IQR)	1.23 (1.17, 1.29)	1.23 (1.17, 1.29)	1.22 (1.16, 1.28)	1.21 (1.15, 1.27)	1.19 (1.10, 1.24)	1.19 (1.13, 1.26)	1.18 (1.11, 1.24)	1.17 (1.10, 1.24)
Ankle-brachial index < 0.9 n (%)	49 (0.4)	31 (0.2)	19 (0.4)	27 (1.4)	28 (0.2)	12 (0.1)	9 (0.5)	7 (0.9)
CACS ≥ 100	2657 (19.1)	1647 (16.1)	552 (21.1)	458 (35.3)	908 (6.1)	590 (4.9)	190 (8.9)	128 (15.9)
Any carotid plaque (≥ 1 vessel)	8928 (61.6)	6342 (60.0)	1590 (62.6)	996 (71.2)	7536 (49.1)	5888 (47.9)	1153 (52.2)	495 (58.6)

## Results

Prediabetes was detected in 3575 cases by IFG only (14.0%) and in 766 cases by elevated HbA1c only (3.0%). In 463 individuals (1.8%), prediabetes was detected both by elevated fasting glucose (IFG) and by an elevated HbA1c level. Of the 2282 SCAPIS participants classified as having diabetes, 1440 had known diabetes (5.6%) and 842 (3.3%) had diabetes not yet detected prior to the baseline visit in SCAPIS. Baseline characteristics by glycaemic status and sex are shown in Table 1.

The frequency of atherosclerosis in the 11 most proximal coronary segments in men and women by glycaemic status in the study population is shown in Fig. 2a, b. The heat map refers to the frequency of any form of atherosclerosis.

Figure 3a shows the distribution of CACS in the three glycaemic categories stratified by sex. The corresponding data for the prevalence of plaque in the carotid arteries are shown in Fig. 3b.

Figure 4 shows the prevalence of CCTA-detected atherosclerosis, carotid artery plaque and peripheral artery disease in SCAPIS participants stratified by glycaemic status in four categories: normoglycaemia, prediabetes, previously undetected diabetes or known diabetes.

Ordinal and logistic regressions analyzing associations between glycaemic status in four categories and CACS, carotid artery plaque and peripheral artery disease, respectively, are shown in Table 2.

**Table 2:** Ordinal and logistic regressions analyzing associations between glycaemic status in four categories and CACS, carotid artery plaque and peripheral artery disease

Variable	*n*	OR	95% C.I	OR^a^	95% C.I^a^	*n*	OR	95% C.I	OR^a^	95% C.I^a^	*n*	OR	95% C.I	OR^a^	95% C.^a^
	CACS					Carotid artery plaque					ABI < 0.9				
Model I Independent: Glycaemic status (ref normoglycemia)	27.916					28.802					24.775				
Known diabetes		**2.65**	2.38–2.96	**2.53**	2.27–2.84		**1.49**	1.34–1.65	**1.37**	1.23–1.53		**10.40**	4.30–25.15	**10.05**	4.06–24.85
Previously undetected diabetes		**1.70**	1.47–1.96	**1.68**	1.45–1.93		**1.16**	1.01–1.33	1.14	0.99–1.31		**7.42**	3.27–16.86	**7.02**	3.06–16.12
Prediabetes		**1.24**	1.16–1.33	**1.23**	1.15–1.31		1.06	1.00–1.13	1.05	0.99–1.12		**2.85**	1.30–6.27	**2.70**	1.22–5.99
Model II Independent: HbA1c	27.796					28.680					25.550				
HbA1c^b^		**1.04**	1.03–1.04	**1.04**	1.03–1.04		**1.02**	1.02–1.02	**1.02**	1.01–1.02		**1.05**	1.04–1.07	**1.05**	1.03–1.07
Model III Independent: Diabetes duration (years)	1.103					1.176					1.044				
Diabetes duration		**1.03**	1.02–1.04	**1.03**	1.02–1.04		1.01	1.00–1.02	**1.01**	1.00–1.03		0.98	0.91–1.05	0.97	0.89–1.07

Bold values are statistically significant

Associations between CACS, Carotid artery plaque and ABI, and glycaemic status (four categories), HbA1c and diabetes duration, respectively. CACS was categorized into four groups (0, 1–99. 100–399, ≥ 400). Carotid artery plaque was categorized into three groups (no plaque, one vessel, both vessels). Ankle-brachial index was categorized into ABI < 0.9 vs ≥ 0.9. All regression models were adjusted for age, sex, smoking and site

^a^Sensitivity analysis where individuals with previous MI, CABG, PCI were excluded (n = 693)

^b^HbA1c regardless of glycaemic status. All significant p-values remained statistically significant (sensitivity analyses excluded) after controlling for the False Discovery Rate as described by Benjamini and Hochberg

In short, known diabetes, undetected diabetes or prediabetes increased the likelihood of belonging to a higher category of CACS, plaque in the carotid arteries and peripheral artery disease compared to individuals with normoglycaemia. In a sensitivity analysis excluding individuals with previous myocardial infarction, coronary bypass grafting, or percutaneous coronary intervention (n = 693), the results for CACS and plaque in the carotid arteries were virtually unchanged (Table 2). Furthermore, higher HbA1c and longer diabetes duration was also associated with the odds of belonging to a higher CACS and carotid artery plaque group.

## Discussion

In this study, CCTA and carotid ultrasound were used in a large random sample of the general population to determine atherosclerotic burden in individuals with normoglycaemia compared with individuals with prediabetes or diabetes. We found that subclinical atherosclerosis in the coronary arteries, as well as plaque in each carotid artery, was more common in people with diabetes and prediabetes compared to normoglycaemic participants in both men and women. In addition, significant atherosclerotic burden in the coronary arteries, as measured by SIS, was more common in people with diabetes and prediabetes than in normoglycaemic individuals. When the diabetes category was divided into undetected diabetes and known diabetes, we found that the atherosclerotic burden in the coronary and carotid arteries in the undetected diabetes category was midway between the prediabetes category and patients with known diabetes, as shown in Fig. 4. This finding may seem intuitively expected, but to our knowledge has never been shown in a large population-based study.

The prevalence of peripheral artery disease was very low. Nevertheless, diabetes was clearly associated with an ABI < 0.9 when adjusted for age, sex, site, and smoking (Table 2). It has been argued that the validity of the ABI may be reduced in diabetes, as ankle pressure may be increased by medial arterial calcification and arterial stiffness, which are more common in diabetes. However, most guidelines still recommend the use of ABI measurements in routine diabetes care because the associations between ABI and cardiovascular and all-cause mortality are similar in people without and with diabetes [18].

It is generally believed that the effects of worsened glycaemic control on increased risk of cardiovascular disease (CVD) are quite long lasting. For example, observational studies showing increased CVD risk in people with prediabetes have an observation period of up to 10 years [19, 20].

Our population-based results suggest a clear correlation between atherosclerosis and any level of increasing dysglycaemia in two important vascular beds. Although it is difficult to draw firm conclusions about time-dependent associations from cross-sectional data, our results suggest a direct link between worsening glycaemic control and increasing prevalence of atherosclerosis.

### Comparison with other studies

Several population-based imaging studies have used CT to quantify CACS as a surrogate marker of atherosclerosis [21–24]. However, no estimates of coronary artery stenosis can be derived from these studies. Previous data on the prevalence of coronary atherosclerosis in the general population are scarce.

Type 2 diabetes is a known risk factor for macrovascular disease and subclinical organ damage, e.g., defined by presence of calcification of the coronary arteries and plaque in the carotid arteries. However, with regards to prediabetes, the results of the different studies vary. Depending on which definition of prediabetes was used, IGT, IFG or elevated HbA1c, findings have varied with respect to associations with coronary and carotid artery calcification. In several studies, prediabetes was associated with increased CACS compared with normoglycaemic participants [3–7]. However, in one study where prediabetes was defined by IFG value only, there was no such association after adjustment [25].

Our results essentially confirm and extend previous observations with respect to diabetes and CACS. What does this study add to what is already known about this topic? First, what is new about our study is that CCTA was used to define coronary artery disease and ultrasound was used to detect plaques in the carotid artery in a large cohort of European ancestry. CCTA provides information on the precise location of coronary plaques, the degree of stenosis, the presence of non-calcified plaques and plaque morphology. We confirm the results of the CONFIRM study, in which CCTA was used to detect and compare coronary atherosclerosis in patients with diabetes compared to diabetic individuals [26]. We can also confirm the results of a smaller South Korean study where CCTA detected atherosclerosis in the coronary arteries and IFG was used to define prediabetes [27]. Second, within the diabetes category, both patients with previously undetected diabetes and those with diabetes had more calcifications in the coronary arteries than the prediabetes group. Third, to the best of our knowledge, we present population based CCTA data comparing the prevalence of atherosclerosis in patients with previously undetected diabetes and study participants with known diabetes and prediabetes. We found a stepwise increase of prevalence of atherosclerosis in the coronary arteries from participants with normoglycaemia, to those with prediabetes, and further to previously undetected diabetes and finally to those with known diabetes, presumably due to longer exposure to dysglycaemia. Data on the association between different stages of dysglycaemia and atherosclerotic burden in the coronary arteries are relevant because we know from previous, non-population-based studies in this area that large plaque burden [28] and severity of coronary artery disease [29] substantially predict future cardiovascular events and mortality.

A similar study from MESA, which used both CACS and carotid ultrasound, looked at the differences between people with metabolic syndrome or diabetes compared with participants who had neither metabolic syndrome nor diabetes. Interestingly, follow-up data from this study suggest that CACS screening strongly stratifies CVD event risk in individuals with metabolic syndrome and diabetes [30]. Furthermore, follow-up data from CONFIRM showed that in patients with diabetes, coronary atherosclerosis according to CCTA was associated with higher all-cause mortality and CVD risk, and significantly higher than in nondiabetic subjects. More importantly, patients with diabetes without coronary atherosclerosis had a comparable risk to nondiabetic subjects [31].

Against this background, our data, which provide deeper insights into the atherosclerotic burden at different stages of dysglycaemia, may have practical implications for future screening strategies for patients with dysglycaemia.

### Strengths and limitations

The greatest strength of the present study is that both the degree of coronary atherosclerosis and carotid plaque were measured in a large population-based sample. The strengths of this study are, however, balanced by some limitations. Although we successfully scanned 86% of the SCAPIS population and obtained high-quality CCTA images, a more aggressive imaging strategy could be used in a real-world screening setting [10]. Additionally, in this study, we used visually detected plaques in the carotid arteries as a measure of carotid atherosclerosis according to the Mannheim consensus [16]. However, other studies in this field have used total plaque area in mm^2^ [32]. Furthermore, we do not have data to categorise the degree of stenosis in the carotid arteries. Regarding external validity, we know from the pilot study that low socioeconomic status was associated with a lower participation rate [33]. We also observed an association between living in an area with low socioeconomic status and increased coronary artery calcification [34]. However, this bias would most likely lead to an underestimation of the association between dysglycaemia and subclinical atherosclerosis in our study.

Finally, prediabetes was defined in this study by IFG or an elevated HbA1c level [12, 13], as we did not have 2 h post-load plasma glucose data from an oral glucose tolerance test to identify individuals with IGT. However, it is reasonable to assume that the elevated 2 h plasma glucose level is likely to provide a more accurate prediction of future cardiovascular events than prediabetes defined by fasting glucose and HbA1c [35].

## Conclusions

The results of the present study show that asymptomatic atherosclerotic burden increases with increasing dysglycaemia in major vascular beds, as the coronary arteries, in a large population-based cohort. The data may have future implications for screening strategies and tailored preventive interventions for people with dysglycaemia.

## Data Availability

The data underlying this manuscript cannot be shared publicly for legal regulations related to the privacy of individuals that participated in the study. The data will be shared on reasonable request to the corresponding author.

## Abbreviations

ABI: Ankle-brachial index
CABG: Coronary artery bypass graft
CACS: Coronary artery calcium score
CCTA: Coronary computed tomography angiography
CRP: C-reactive peptide
CVD: Cardiovascular disease
IFG: Impaired fasting glucose
IGT: Impaired glucose tolerance
MI: Myocardial infarction
PCI: Percutaneous coronary intervention
SCAPIS: Swedish CArdioPulmonary bioImage Study
SIS: Segment involvement score
